# Genome Sequence of *meso*-2,3-Butanediol-Producing Strain *Serratia marcescens* ATCC 14041

**DOI:** 10.1128/genomeA.00590-14

**Published:** 2014-06-19

**Authors:** Lixiang Li, Yu Wang, Kun Li, Fei Su, Cuiqing Ma, Ping Xu

**Affiliations:** a State Key Laboratory of Microbial Metabolism, Shanghai Jiao Tong University, Shanghai, China; b State Key Laboratory of Microbial Technology, Shandong University, Jinan, China

## Abstract

*Serratia marcescens* strain ATCC 14041 was found to be an efficient *meso*-2,3-butanediol (*meso*-2,3-BD) producer from glucose and sucrose. Here we present a 5.0-Mb assembly of its genome. We have annotated 4 coding sequences (CDSs) for *meso*-2,3-BD fermentation and 2 complete operons including 6 CDSs for sucrose utilization.

## GENOME ANNOUNCEMENT

As an important commodity chemical, 2,3-butanediol (2,3-BD) can be used as the starting material for some bulk chemicals such as methyl-ethyl-ketone, gamma-butyrolactone, and 1,3-butadiene (1–3). It also has various potential applications in the manufacture of food additives, cosmetics, drugs, and explosives (1). With a heating value of 27,200 J/g, 2,3-BD compares favorably with ethanol (29,100 J/g) for using as a liquid fuel or fuel additive (3). In addition, *meso*-2,3-BD can also be used to produce renewable polyesters, such as semi-aromatic polyesters for coating applications (4, 5). Microbial production of 2,3-BD has a long history, and various bacteria, such as *Serratia marcescens*, *Bacillus licheniformis*, and *Klebsiella pneumoniae*, were used to produce 2,3-BD (2, 6–9). However, among the native 2,3-BD producers, only *S. marcescens* was reported to be able to produce *meso*-2,3-BD as the major product (2). Our unpublished results indicated that *S. marcescens* strain ATCC 14041 is an efficient *meso*-2,3-BD producer with high productivity (>2 g liter^−1^ h^−1^) and high concentration (>100 g liter^−1^). It was found that the purity of *meso*-2,3-BD decreased in the fermentation course from 99% to 96%. This might be caused by other enzymes that could catalyze acetoin to (2*R*,3*R*)-2,3-BD or (2*S*,3*S*)-2,3-BD. To eliminate by-product formation and to enhance the purity of *meso*-2,3-BD, genetic modification is desirable. Genome sequencing of strain ATCC 14041 will be of great help in this regard.

Here, we present the draft genome sequence of strain ATCC 14041, which was obtained using the Illumina HiSeq 2000 system. The reads for strain ATCC 14041 were assembled into 197 contigs using VELVET (10). The genome annotations were performed by the RAST server (11). The functional descriptions were determined using the Clusters of Orthologous Genes database (12). The G+C content was calculated using the genome sequence.

The draft genome sequence of strain ATCC 14041 consists of 5,047,446 bases with a G+C content of 59.8%. According to the annotation of the RAST system, there are 4,649 protein-coding sequences (CDSs) and 109 RNAs in the genome, among which 2,678 CDSs (58%) were assigned putative biological functions. A total of 574 subsystems were determined using the RAST server in the genome, and this information was used to construct the metabolic network by the RAST system. There are key enzymes for utilization of fructose, mannose, sorbitol, glycerol, and xylitol in strain ATCC 14041, indicating that strain ATCC 14041 may have a wide substrate spectrum. The sequence contains operons and key coding genes for 2,3-BD formation. There is a gene encoding glycerol dehydrogenase which could catalyze (3*R*)-acetoin to (2*R*,3*R*)-2,3-BD (13). These results could provide further insights into production of 2,3-BD. Based on carbohydrate metabolism analysis, six CDSs for the metabolism of sucrose were annotated in two independent gene clusters. This might result in the high efficient utilization of sucrose to produce 2,3-BD.

### Nucleotide sequence accession number.

The whole-genome shotgun projects have been deposited at DDBJ/EMBL/GenBank under accession no. JGVB00000000 for strain ATCC 14041. The version described in this paper is the first version.
